# Functional and Pharmacological Comparison of Human, Mouse, and Rat Organic Cation Transporter 1 toward Drug and Pesticide Interaction

**DOI:** 10.3390/ijms21186871

**Published:** 2020-09-19

**Authors:** Saskia Floerl, Annett Kuehne, Yohannes Hagos

**Affiliations:** PortaCellTec Biosciences GmbH, 37075 Goettingen, Germany; floerl@portacelltec.de (S.F.); kuehne@portacelltec.de (A.K.)

**Keywords:** solute carrier (SLC) family, OCT1, SLC22A1, species differences, drugs, pesticides

## Abstract

Extrapolation from animal to human data is not always possible, because several essential factors, such as expression level, localization, as well as the substrate selectivity and affinity of relevant transport proteins, can differ between species. In this study, we examined the interactions of drugs and pesticides with the clinically relevant organic cation transporter hOCT1 (SLC22A1) in comparison to the orthologous transporters from mouse and rat. We determined K_m_-values (73 ± 7, 36 ± 13, and 57 ± 5 µM) of human, mouse and rat OCT1 for the commonly used substrate 1-methyl-4-phenylpyridinium (MPP) and IC_50_-values of decynium22 (12.1 ± 0.8, 5.3 ± 0.4, and 10.5 ± 0.4 µM). For the first time, we demonstrated the interaction of the cationic fungicides imazalil, azoxystrobin, prochloraz, and propamocarb with human and rodent OCT1. Drugs such as ketoconazole, clonidine, and verapamil showed substantial inhibitory potential to human, mouse, and rat OCT1 activity. A correlation analysis of hOCT1 versus mouse and rat orthologs revealed a strong functional correlation between the three species. In conclusion, this approach shows that transporter interaction data are in many cases transferable between rodents and humans, but potential species differences for other drugs and pesticides could not be excluded, though it is recommendable to perform functional comparisons of human and rodent transporters for new molecular entities.

## 1. Introduction

Numerous hydrophilic compounds require membrane transporters to surmount the plasma membrane of cells. Members of the ATP-binding cassette (ABC) as well as transporters belonging to the solute carrier (SLC) transporter superfamily facilitate the cellular entry or exit of small organic molecules. The driving force for ABC transporter-mediated efflux is provided by ATP hydrolysis, classifying the ABC transporters as primary active. The SLC transporters translocate their substrate through the plasma membrane by electrochemical gradients. Thus, they are secondary or tertiary active transporter. The organic cation transporter 1 (OCT1) is the first member of the SLC22 subfamily (SLC22A1). In 1994, rOct1 was initially identified from rat kidney and encoded 556 amino acids [1,2]. In the following years, several mammalian OCT1 orthologs from human, mouse and rabbit were identified [3,4]. Human OCT1 consists of 554 amino acids and shares 78% sequence identity with both mouse and rat Oct1. Human OCT1 is highly expressed in the liver [3,4], where it is located in the sinusoidal membrane of hepatocytes [5]. In rodents, Oct1 is expressed not only in the liver but also highly in the kidney, small intestine, and lung [6]. In the small intestine, OCT1 is localized at the luminal membrane of enterocytes [7,8], in contrast to the basolateral expression of OCT1 in hepatocytes. In the human liver, the highest expression of membrane transporters was demonstrated for hOCT1 [9]. OCT1 mediates the uptake of cationic substrates from the sinusoid into hepatocytes and contributes to the first step of hepatic excretion of endogenous as well as exogenous cationic compounds. In humans, OCT1 enables the reabsorption of organic cations from primary urine, unlike rodent Oct1, which is expressed on the basolateral membrane of proximal tubule cells [10]. As a multi-specific transporter, OCT1 translocates structurally different endogenous as well as exogenous substrates such as choline, corticosterone, acetylcholine, guanidine, and drugs such as metformin, atropine, ranitidine, cisplatin derivates, sumatriptan, morphine, as well as toxins, such as aflatoxin B1, monocrotaline, and ethidiumbromide [8,9,11,12].

Human OCT1 is one of around ten SLC and ABC transporters selected by the Food and Drug Administration (FDA) and European Medicines Agency (EMA) [13] to be tested as part of drug approval, because of their clinical relevance as drug and toxin transporters and the possible involvement in drug–drug interactions (DDI). The initial toxicity as well as pharmacokinetic studies in early drug development are accomplished mainly in laboratory animals, particularly in rodents. Generally, mice and rats are the species of first choice in drug development, since their organisms are very similar to that of humans in many respects, but side effects, such as nephrotoxicity or hepatotoxicity, which have not been observed in animal experiments on rodents occur repeatedly in humans. Therefore, the data generated from animals could not always be extrapolated to humans. For example, troglitazone showed severe hepatotoxic effects in man that had not been observed in regulatory animals [14], which could be due to adverse effects in drug-induced liver injury caused by species-specific susceptibilities [15]. The reasons for the species differences also include physiological parameters in which mice and humans differ, such as body weight and organ-specific excretionn processes in the kidney and the liver. Drugs excreted via the liver encounter different physiological parameters, such as species differences in biliary excretion. The bile flow in rats and mice is 90 and 100 mL/day/kg, respectively, whereas the bile flow in humans is 5 mL/day/kg [16]. Anatomically, humans and mice can store the bile in the gallbladder, whereas rats do not have a gallbladder and therefore continuously excrete the bile into the intestine. The biliary excretion of endogenous as well as exogenous compounds, such as drugs, is dependent on the activity of SLC and ABC transporters. Membrane transporters play a pivotal role in the absorption, distribution, metabolism, and elimination (ADME) of drugs. Therefore, it is crucial to compare the data generated from rodents with humans in in vitro assays to evaluate the impact of the transporter to DDI for humans during the potential use in therapy. Dresser et al. demonstrated species-dependent differences in the interaction of OCT1 with n-tetraalkylammonium derivatives [6,17].

The aim of this project was to figure out as to what extent species differences play a role in the transport function of OCT1. For that, we carried out interaction studies of chemical substances with hOCT1, rOct1, and mOct1 under the same conditions. As the first part of this project, we compared the affinity of the known substrate 1-methyl-4-phenylpyridinium (MPP) and the inhibition data of the known inhibitor decynium22 toward hOCT1 with the data of the orthologous mouse and rat Oct1. While in the second part, we correlated the hOCT1 interaction data of several drugs and also of pesticides with the data of mouse and rat Oct1. In this context, the involvement of the SLC transporter OCT1 in the interaction with pesticides ought to be examined to compare a broad spectrum of chemical entities that humans are exposed to. The interaction of a vast number of drugs with OCTs is intensively investigated. In contrast, the interaction of pesticides with SLC transporters, and particularly with OCTs, is barley examined, despite the increasing interest from regulatory authorities and producers of pesticides. In this project, we elucidated the impact of pesticides in the inhibition of OCT1, since these compounds could be involved in pesticide–drug interactions.

Hundreds of pesticides are used worldwide in agricultural holdings and large agricultural industries. To avoid or minimize the exposure of employees and consumers to pesticides, there are internationally harmonized definitions of the Maximum Residue Level (MRL) of pesticides in foodstuffs as well as the tolerable daily intake for humans as Acceptable Daily Intake (ADI). These parameters help to control the potential chronic toxicity by continued intake of foodstuffs contaminated with pesticides. The ADI is obtained by feeding rats certain amounts of pesticides through their food for a very long time. If the rats tolerate this chemical without any health consequences, the daily allowable dose (ADI) is achieved in mg of active substance per kg of body weight per day. For safety reasons, the permitted daily dose for humans should be only 1% of the permitted daily dose for rats. Nevertheless, the pharmacokinetics or ADME processes of several pesticides in humans and the involvement of membrane transporters in the liver and kidney, which are crucial for the absorption, elimination, and DDI, are not well understood. Therefore, the additional intention of this work was to expound the interaction of human OCT1 with pesticides and to correlate with the mouse and rat Oct1 interaction. Important parameters suh as ADI are generated by the exposure of animals to these chemical entities to prevent the potential pesticide toxicity to humans. Hence, it is very important to compare the interaction of the membrane transporter with pesticides across different species, particularly with rodents.

## 2. Results

### 2.1. Functional Characterization of Human, Mouse, and Rat OCT1

**Figure 1:**
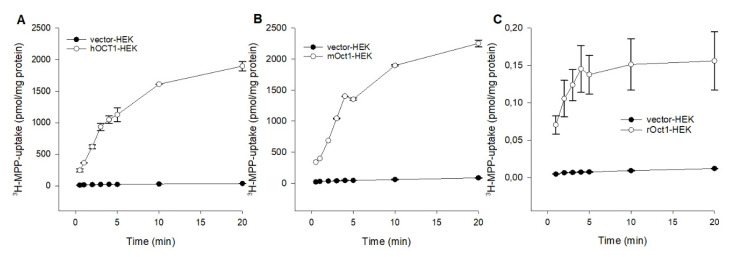
Time dependent uptake of 3H-MPP in (**A**) hOCT1-, (**B**) mOct1-, and (**C**) rOct1-HEK293 cells. Human, mouse, and rat organic cation transporter 1 (OCT1) transfected HEK293 cells were incubated for increasing time points at 37 °C in the presence of labeled 3H-1-methyl-4-phenylpyridinium (MPP) (2 nM), in case of hOCT1 and mOct1 the concentration was filled up to 10 µM with unlabeled MPP. Each data point represents the mean of two or three independent experiments ± average deviation. Experiments were carried out in triplicates.

To compare the fundamental functional characteristics of human, mouse, and rat OCT1 under comparable conditions in stable transfected HEK293 cells, initial hOCT1-, mOct1-, and rOct2-mediated time-dependent uptake experiments were performed. Using MPP as substrate, the uptake into OCT1-HEK cells was measured over a period of 0.5 to 20 min, as shown in Figure 1. The MPP uptake facilitated by hOCT1, mOct1, and rOct1 increased linear up to 3 min and was saturated at 10 min for all species. Initial 1 min uptake of hOCT1-HEK, mOct1-HEK and rOct-HEK cells was 20.3-, 15.6-, and 14.3-fold higher than the uptake of the control cells; therefore, further MPP uptake experiments were terminated for all OCT1 transporters at 1 min.

**Figure 2:**
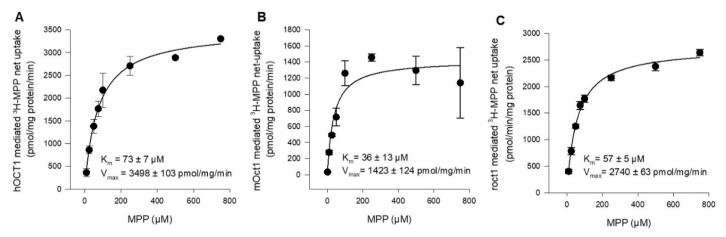
Kinetics of (**A**) hOCT1-, (**B**) mOct1-, and (**C**) rOct1-mediated 3H-MPP transport. Human, mouse, and rat OCT1-transfected HEK293 cells were incubated for 1 min at 37 °C in the presence of labeled (2 nM) and increasing concentrations of non-labeled MPP. Net uptake was fitted to the Michaelis–Menten equation to obtain the affinity constant *K*_m_ and maximum transport velocity *V*_max_ by non-linear regression analysis using Sigma Plot 13.0 software. Each data point represents the mean of two independent experiments ± average deviation. Experiments were carried out in triplicates.

To determine and compare the affinity of hOCT1, mOct1, and rOct1 in the same expression system and under the same experimental conditions, concentration-dependent MPP uptake was conducted. In transporter-transfected and vector-transfected HEK293 cells, we measured the uptake of MPP in a transport buffer containing 2 nM [^3^H]-labeled MPP in the presence of increasing concentrations of non-labeled MPP. The *K*_m_ value of hOCT1, mOct1, and rOct1 were determined to be 73 ± 7 µM, 36 ± 13 µM, and 57 ± 5 µM, as shown in Figure 2A–C, respectively. The substrate turnover calculated as the *V*_max_ value of mOct1 (1423 ± 124 pmol/mg/min) and rOct1 (2740 ± 63 pmol/mg/min) was inconsiderably (<2.5-fold difference) lower than the *V*_max_ value of hOCT1 (3498 ± 103 pmol/mg/min) for MPP.

**Figure 3:**
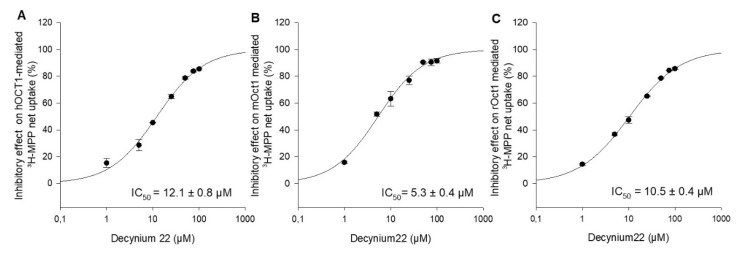
Inhibitory effects of decynium22 on (**A**) hOCT1-, (**B**) mOct1-, and (**C**) rOct1-mediated ^3^H-MPP transport in stable transfected HEK293 cells. Uptake of MPP at Km value was measured in the presence of increasing concentrations of decynium22 (1–100 µM). Each data point represents the mean inhibitory effect (%) calculated from the net-uptake of two independent experiments ± average deviation. Each experiment was carried out in triplicates. IC_50_ values were calculated by sigmoidal 3Hill analysis using Sigma Plot 13.0 software.

Decynium22 is a well-known, high-affinity inhibitor of OCT1, OCT2, and OCT3 [2,17,18]. To our knowledge, there are no systematical studies under the same conditions to evaluate the concentration-dependent inhibition of hOCT1, mOct1, and rOct1 by decynium22. For further functional characterization and comparison of the three species, the inhibitory potential of the increasing decynium22 concentrations on OCT1-mediated uptake of MPP was measured, and the IC_50_ value of decynium22 for hOCT1, mOct1, and rOct1 was calculated to be 12.1 ± 0.8 µM, 5.3 ± 0.4 µM, and 10.5 ± 0.4 µM (Figure 3).

### 2.2. Comparison of the Interaction of hOCT1, mOct1, and rOct1 with Drugs and Pesticides

After the basic functional validation, a comparison of hOCT1, mOct1, and rOct1 interaction with fifteen drugs from different classes of compounds used for specific therapeutic targets as well as nine pesticides frequently applied in agricultural industries were evaluated.

Inhibition assays towards human OCT1, mouse Oct1, and rat Oct1 were conducted to compare the species-dependent interaction of ketoconazole, clonidine, verapamil, quinine, elacridar, quinidine, procainamide, ritonavir, ranitidine, zosuquidar, metformin, amiodarone, cimetidine, cyclosporine A, and reserpine. The OCT1-facilitated MPP uptake was inhibited in the presence of 10 or 100 µM of each drug. Ketoconazole, clonidine, verapamil, quinine, elacridar, quinidine, and procainamide inhibited the transport activity of hOCT1, mOct1, and rOct1 at 100 µM by more than 50%. The seven above-mentioned drugs showed high, comparable, and species-independent inhibitory effects on hOCT1, mOct1, and rOct1, as depicted in Table 1. Slight differences at a very low level were observed, for example, for ritonavir and zosuquidar. Ritonavir demonstrated at 100 µM a reduction in rOct1- and hOCT1-mediated MPP uptake to 78% and 61%. In contrast, ritonavir stimulated at 100 µM the mOct1 transport activity by up to 16%. Zosuquidar revealed the low inhibition of the hOCT1-facilitated transport of MPP and no inhibition of rOct1 but a slight (23%) stimulation of the MPP uptake by mOct1. However, neither of the drugs showed clear interaction differences between human, mouse, and rat OCT1 transport activity.

The following pesticides were examined to elucidate their inhibitory potential on the OCT-mediated MPP uptake: imazalil, propamocarb, azoxystrobin, prochloraz, atrazin, amitraz, glyphosate, imidacloprid, and paraquat. The highest inhibition of OCTs was observed with imazalil, propamocarb, and azoxystrobin. They reduced the transporter-mediated uptake of MPP in the presence of 100 µM by 50% or more. The other pesticides showed no or only slight inhibitory effects. Some pesticides showed stimulation of OCT1-mediated MPP uptake, as summarized in Table 2. None of the pesticides demonstrated a clear differential species-dependent interaction within human, mouse, and rat OCT1 transport activity.

**Figure 4:**
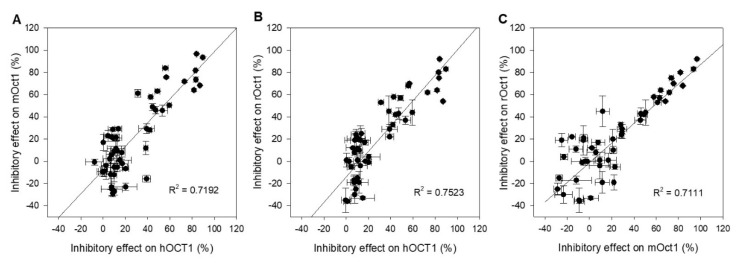
Scatter plot analysis correlating inhibitory effects of (**A**) hOCT1 and mOct1, (**B**) hOCT1 and rOct1, and (**C**) mOct1 and rOct1. The correlation coefficient *R*^2^ value for all three combinations is higher than 0.7, indicating a good correlation between species. Data points represent mean and standard deviation of one individual experiment. Mean values in detail are presented in Table 1 and Table 2.

Correlation analyses were carried out to visualize the interaction studies performed with drugs and pesticides toward hOCT1-, mOct1-, and rOct1-transfected HEK293 cells. The inhibitory effect of fifteen drugs and nine pesticides at both concentrations was plotted to evaluate interaction outcome of two transporters of different species. The Figure 4A–C present the functional correlation of hOCT1 versus mOct1, hOCT1 versus rOct1, and mOct1 versus rOct1. The correlation coefficient R^2^ of all three plots was higher than 0.7, representing a good functional correlation of OCT1 within the species.

Furthermore, to compare the drug and pesticide interaction of human OCT1 with other members of organic cation transporters belonging to the SLC22A and SLC47A (hOCT2 and hMATE1) families, additional inhibition studies with drugs and pesticides were performed. The hOCT2-mediated MPP as well as hMATE1-mediated metformin uptake was inhibited at 10 and 100 µM of fourteen cationic drugs and nine mainly cationic pesticides. The inhibition studies were measured in stable transfected HEK293 cells at comparable conditions. As shown in Table 3, decynium22 revealed transporter-dependent high inhibition down to 3% to 10% of metformin or MPP uptake by hMATE1, hOCT1, and hOCT2. The highest inhibition of hOCT1, hMATE1, and hOCT2 at 100 µM of pesticides was achieved with imazalil to 16%, 17%, and 39% remaining transporter activity.

**Figure 5:**
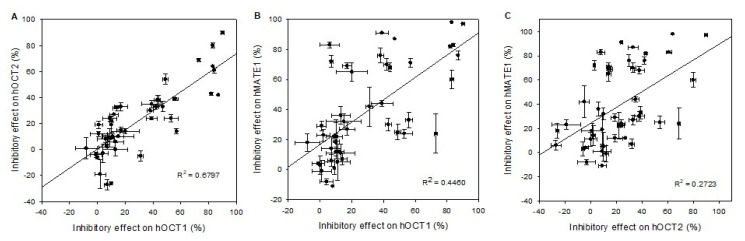
Scatter plot analysis correlating inhibitory effects of (**A**) hOCT1 vs. hOCT2, (**B**) hOCT1 vs. hMATE1, (**C**) and hOCT2 vs. hMATE1. The correlation coefficient *R*^2^ value for the combination hOCT1 vs. hOCT2 is near 0.7, indicating a good correlation between cation transporters, while the combination hOCT1 vs. hMATE1 shows only an R^2^ value of 0.45 and for hOCT2 vs. hMATE1 *R*^2^ = 0.27. Data points represent mean and standard deviation of one individual experiment. Mean values are presented in detail in Table 3 and Table 4.

The correlation analyses of the drug and pesticide interaction with hOCT1 versus hOCT2, as plotted in Figure 5A, shows with a correlation coefficient R^2^ of 0.67 a good correlation for the selected compounds. Yet, for a few compounds (e.g., elacridar), there is no clear correlation between hOCT1 and hOCT2 (see Table 3).

Human OCT1 and MATE1 show for a few compounds inhibitory effects at the same level, e.g., imazalil at a very high level (83%), and amitraz at a very low level (12%) (see Table 4). However, the inhibitory effects of a large number of the compounds do not reveal a functional correlation of hOCT1 and hMATE1, as presented in Figure 5B. The calculated functional correlation coefficient R^2^ was 0.45, which is remarkably lower than the coefficient between the OCT1 species or between hOCT1 and hOCT2.

## 3. Discussion

Laboratory animals are indispensable tools in the initial preclinical drug development and evaluation of the pharmacokinetics of new molecular entities (NMEs). In vivo, they deliver pivotal data in terms of toxicity and achievement of the therapeutic target as well as ADME. The parameters received from animal experiments reflect the systemic performance of the compound after treatment. Additional in vitro experiments are crucial to address specific interactions of NME with metabolizing enzymes, target proteins, permeability of the plasma membrane as well as transporter proteins, which mediate the intake or the release of the compounds for the cells. Therefore, the potential species differences should also be considered in the in vitro experimental setups.

The objective of this study was to compare the drug and pesticide interaction with human and rodent organic cation transporter 1 (OCT1; SLC22A1). There are several studies demonstrating the interaction of drugs with human, mouse and rat OCT1. Nevertheless, the direct comparison of the data is difficult, since most of the results are generated with different expression systems, substrates, and experimental conditions. Consequently, in our study, functional characterization and validation of the stable in HEK293 cell transfected human, mouse, and rat OCT1 was carried out, starting with the time-dependent MPP uptake, where all three transporters were saturated after 10 min and the linear uptake extended to 3 min. In the first time-dependent functional evaluation of rOct1 and hOCT1 by injecting cRNA in *Xenopus laevis* oocytes, a linear uptake of ^14^C-TEA of 90 min and 120 min ^3^H-MPP uptake was observed [1]. The substantial difference on the linearity of the uptake in *X. laevis* oocytes and HEK293 cells could be the expression of the OCTs in the plasma membrane, which is not comparable. In several studies, the group of Mladen Tzvetkov demonstrated the linear uptake of several compounds in OCT1-expressing HEK293 cells within 2 min [9,11,22]. The affinity of the OCT1 transporter different species for specific small molecules could differ within the same expression system, as demonstrated by Dresser et al. They compared the interaction and affinity of n-tetraalkylammonium derivates with human, mouse, rat, and rabbit OCT1 expressed in *X. laevis* oocytes and showed 4-fold higher affinity of mOct1 to TBA than the hOCT1 [17]. Therefore, it was very important to determine the affinity of the three transporters to MPP (*K*_m_ values) under the same conditions. In our study, the *K*_m_ values of human and rodent OCT1 were in a comparable range. Nevertheless, the affinities for mOct1 and rOct1 were slightly higher than the affinity for hOCT1. However, these differences were not significant according to Student’s *t*-test (*p* > 0.05).

This indicates also a study of Gründemann and colleagues [23], where the *K*_m_ value of hOCT1-HEK293 cells was determined to be 32 µM, which is 2.2-fold lower than was observed in our study, even though the experimental conditions were comparable except for the fact that the HBSS buffer used in this study contained bicarbonate. *K*_m_ values of 10 µM and 5.6 µM were determined for mOct1 and rOct1 expressed *X. laevis* oocytes [24,25]. For further validation of hOCT1-, mOct1-, and rOct1-expressing HEK293 cells, the concentration-dependent inhibitory potential of the well-known OCT inhibitor [6] decynium22 was determined and the *K*_m_ values were calculated. Similarly, the IC_50_ values of decynium22 for hOCT1 and rOct1 were almost the same and mOct1 exhibited 2-fold higher affinity for decynium22 than the human and rat OCT1. In another study, decynium22 inhibited the MPP uptake in isolated rat hepatocytes as well as in hOCT1-expressing *X. laevis* oocytes, with IC_50_ values of 1.4 and 4.7 µM, respectively [2,26]. The difference on the substrate and inhibitor selectivity as well as affinity with in the orthologues OCT1 transporter could be the specific amino acid variation within the amino acid sequence. As very well discussed by Wright and Dantzler [27] and demonstrated by mutation analysis and the replacement of aspartate 475 to glutamate (D475E) in rOct1 amino acid sequence, the affinity for methylnicotinamide, tetraethylammonium (TEA), and choline increased by 4-, 8- and 15-fold, respectively. In contrast, the affinity of the mutant D475E rOct1 for MPP remained unchanged in comparison to the wild-type rOct1 [28]. Several studies demonstrated numerous hOCT1 polymorphisms as well as a worldwide genetic variability of hOCT1, indicating specific polymorphisms M420del could lead to loss-of-function. For example, 9% of the Caucasian population possesses OCT1 without functional activity [29]. Nevertheless, several SNPs that prompt a specific amino acid exchange in hOCT1 revealed alternated affinity as well as substrate or inhibitor selectivity [9,11,22].

To elucidate the interaction of drugs and pesticides with human, mouse, and rat OCT1, we performed the inhibition of OCT1-mediated uptake of MPP with two concentrations (10 and 100 µM) for each of the 15 drugs and nine pesticides. Seven drugs showed an inhibitory potential with a reduction of the uptake rate to more than 50%. The highest inhibition for hOCT1 was observed at 100 µM for ketoconazole > clonidine > verapamil > quinine > elacridar > quinidine > procainamide. Other drugs revealed very low inhibitory effects on hOCT1 activity. Nevertheless, most of the inhibitor drugs showed comparable inhibition between hOCT1, mOct1, and rOct1, with only slight variation. The results achieved in this study at 10 µM drug inhibitory potential to hOCT1 in % reflect the published IC_50_ values of 2.6–7.4 µM for ketoconazole, 0.6–23 µM for clonidine, 1–13 µM for verapamil, 3.5–96 µM for quinine, 5–340 µM for quinidine, 15–74 µM for procainamide, and 5–34 µM for ritonavir [8]. Elacridar inhibited the OCT1-mediated MPP uptake down to 69% and 39% remaining transport activity at 10 and 100 µM. In this study, we demonstrate, for the first time, the interaction/inhibition of hOCT1, mOct1, and rOct1 by elacridar (also known as GF 120918), an inhibitor of several ABC-efflux transporters.

Pesticides are, unfortunately, a part of our nutrition. Therefore, the responsible agencies worldwide try to protect the consumers by setting the Maximum Residue Level (MRL). However, the MRL is often exceeded accidentally or intentionally [30,31]. Consumers are continually exposed to pesticides, primarily through residues in foodstuff [32] and by close neighborhood to farms intensively treated with pesticides, which leads to an intake of pesticides through inhalation as well as through the skin by contaminated air. Food safety reports 2014 of the German federal office of consumer protection and food safety as well as the commission of the European community for monitoring of pesticide residues in plant products confirmed that pesticide residues were found to different extent in several foods (vegetables). Glyphosate is the most used pesticide worldwide and 4000 exposures were reported by the US poison center each year. Almost 10% of these cases were intentional (suicide) ingestions [33]. Thousands of accidental and intentional deaths by ingestion of paraquat are also observed. A plasma concentration of 734 µg/mL was determined in a patient who intentionally ingested glyphosate. The half-life of glyphosate is 3.1 h [33].

Glyphosate was found in human urine samples possibly as a result of dietary intake or from occupational use [34]. A urinary excretion study from farm families exposed to glyphosate demonstrated a maximum concentration of 233 µg/mL [35]. Similarly, paraquat, imazalil, azoxystrobin, atrazine, amitraz as well as imidacloprid were excreted and identified in urine [36,37,38,39,40,41]. Prochloraz was not detected in urine but several of its metabolites were, e.g., 2,4,6-trichlorophenoxyacetic acid, which was detected mainly as a glucuronide conjugate [42]. The kidney actively secretes numerous pesticides. Therefore, several transporter proteins expressed in proximal tubule cells could be involved in the active secretion of pesticides. The transport of paraquat by hOCT2-expressing HEK cells was reported recently [21]. The interaction of azoxystrobin, propamocarb, and several other pesticides inhibit the efflux activity of rabbit Abcg2 at the MRL level [43]. However, the MRL in foodstuff show enormous concentration differences. For example, propamocarb MRL in cereals is 0.1 mg/kg while in vegetables, it is 500-fold higher (50 mg/kg). Therefore, we used relatively high concentrations (10 and 100 µM) of the nine pesticides, which are mainly positively charged at the physiological pH, to elucidate their inhibitory potential on human, mouse, and rat OCT1. The choice of the high concentrations of the pesticides for the inhibition of OCT1-mediated MPP uptake enabled us to directly compare the inhibitory potential of each pesticide to OCT1 activity. The highest species-independent inhibition of OCT1 was observed for imazalil, followed by propamocarb > azoxystrobin > prochloraz. The hOCT1-, mOct1-, and rOct1-mediated MPP uptake was decreased between 3% and 75%. The inhibition of OCT1 and OCT2 as well the stimulation of MATE2K by propamocarb was reported by Guéniche et al. (2020), but the study also confirmed that propamocarb is not a substrate of the cation transporter [44]. To our knowledge, up to now, there is no data that showed the excretion of propamocarb in urine. Therefore, OCTs as well as MATE2K are most probably not involved in the renal secretion of propamocarb. Nevertheless, the detection of imazalil, azoxystrobin, and prochloraz metabolites in urine might be an active elimination facilitated by the OCTs as well as by MATEs.

Atrazin, amitraz, glyphosat, imidacloprid, and paraquat showed at 100 µM either a marginal inhibitory effect or a stimulation of OCT1 activity. Several tested compounds, particularly ritonavir, amiodarone, glyphosate and atrazine, demonstrated a stimulatory effect between 16% and 65%. Drug-induced cis-stimulation of the reference substrate uptake was observed previously for various influx as well as for efflux transporters and numerous compounds. Hagos et al. demonstrated 24% to 86% stimulation of OAT3 (SLC22A8) as well as OAT4 (SLC22A11)-mediated estrone sulfate uptake by melphalan, respectively [45]. Irinotecan caused 93% stimulation of estrone sulfate uptake by OATP1B1 (SLCO1B1), as reported by Marada et al. (2015) [46]. The mechanism behind these phenomena is still not clear. One possible explanation is the binding of the compound to a specific site of the transporter which generates a higher turnover for the substrate. The consequence is a higher accumulation of the reference substrate in the cells even at relatively low concentrations. This modulation of the transporter is most probably caused by allosteric effects or cooperativity of specific sites within the transporter.

Chen et al. (2007) reported results comparable to our studies concerning the interaction of paraquat with OCT1 but, in contrast, they demonstrated the transport of paraquat by OCT2, while we did not observe a significant interaction for paraquat with hOCT2. Since these pesticides did not interact significantly with OCTs as well as with MATEs, the renal secretion mediated by the cationic transporter that were examined in this study and are located in the kidney could be excluded. Most of these pesticides interact with several efflux transporters. Therefore, it would need further studies to understand the role of SLC transporters in the renal secretion mechanism of the pesticides. In this study, we evaluated the inhibitory potential of drugs and pesticides to hOCT1-, mOCT1- and rOct1-mediated uptake of MPP. Based on our data, we cannot deny that some of the drugs and pesticides that showed an inhibitory potential are also substrates of the OCTs. For a differentiation between inhibitor and substrate there are several options for further studies: If the substance to be examined is available radioactively or fluorescently labeled, a direct measurement of the accumulation in the HEK cells is possible, but the ultimate method to determine the OCT1-mediated uptake of non-labeled drugs and pesticides is by the HPLC tandem LC–MS/MS method. For a precise understanding of the interaction of the above-mentioned drugs and pesticides, further OCT1-mediated substrate uptake by LS–MS/MS analysis are needed.

In conclusion, the present study elucidated, for 26 structurally different and, at pH 7.4, mainly positively charged compounds, a good functional correlation between human, mouse, and rat OCT1. Additionally, we found substantial inhibitory potential for three of the selected pesticides with OCT1, which was not species dependent. Nevertheless, potential species differences within OCT1 could not be excluded for other drugs and pesticides that were not considered in this study. Hence, for clinically relevant new molecular entities, it is recommended to perform functional in vivo as well as in vitro comparisons of the transport in humans and rodents.

## 4. Materials and Methods

### 4.1. Material

^3^H-MPP (1-Methyl-4-phenylpyridinium iodide) and ^14^C-metformin were purchased from American Radiolabeled Chemicals Saint louis; Missouri, USA. All non labelled chemicals were obtained from Sigma-Aldrich, Darmstadt, Germany. For transfection, the following cDNAs were used: hOCT1 (GeneBank: accession number: NM_003057.2), mOct1 (NM_009202.5), rOct1 (NM_012697.1), hMATE1 (NM_018242.2), and hOCT2 (NM_003058.3). The hOCT1 cloned has the genotype Ser14, Arg61, Cys88, Phe160, Gly401,Met408, Met420 and Gly465, which corresponds to the OCT1*1B allele according to the nomenclature suggested by Seitz et al. [29].

### 4.2. Transfection and Cell Culture

The respective cDNA of the cation transporters has been cloned into the expression vector pcDNA5/FRT. Human embryonic kidney (HEK-293-Flp-In) cells (Invitrogen, Darmstadt, Germany) were transfected using Lipofectamine 2000 (Invitrogen, Darmstadt, Germany) according to the manufacturer’s protocol. Twenty-four hours after transfection, 175 µg/mL hygromycin B was added to the medium to select stable clones. After two to three weeks, single colonies were picked and expanded. The growth medium for stably transfected HEK-293 cells was Dulbecco’s modified Eagle’s medium (DMEM, high glucose) supplemented with 10% fetal bovine serum (Biochrom, Berlin, Geramny), 1% penicillin (10.000 Units/mL)/streptomycin (10 mg/mL). Cell lines were grown in a humidified atmosphere containing 5% CO_2_ at 37 °C.

### 4.3. Transporter Mediated Uptake of Radiolabeled Substrates

For uptake assays, 2 × 10^5^ cells in 0.5 mL growth medium per well were seeded into 24-well plates, coated with poly-D-lysine and cultured for 3 days. Then, growth medium was aspirated and each well was rinsed three times with 0.5 mL incubation buffer (HBSS buffer supplemented with 20 mM HEPES, pH 7.4) and incubated at least 20 min at 37 °C as described previously [47]. For hMATE1, it was necessary to generate an intracellular acidification; therefore, the cells were pre-incubated for at least 30 min in a 30 mM NH_4_Cl containing incubation buffer at pH 7.4 and 37 °C.

The incubation buffer was removed and 200 µL incubation buffer containing radiolabeled and non-radiolabeled substances was added to each well and incubated at 37 °C for 1 min. After incubation, the uptake was terminated by aspirating the reaction mixture and washing the cells three times with 0.4 mL ice-cold PBS buffer. Cells were solubilized with 0.6 mL of 1N NaOH overnight. [^3^H] or [^14^C] content was measured after addition of 2.5 mL scintillation solvent (Roti^®^eco plus, Carl Roth, Karlsruhe, Germany ) in a Beckmann LS6000 scintillation counter.

To determine the affinity (*K*_m_) of MPP as a substrate of organic cation transporter, saturation experiments at initial rate period were performed as determined in time dependency experiments (data not shown). Organic cation transporter transfected HEK and empty vector-HEK cells were incubated for 1 min with 2 nM [^3^H] MPP and increasing concentrations of non-labeled MPP: 1, 10, 25, 50, 100, 250, 500, and 750 µM. Experiments were conducted on at least two separate days. On each day, all experiments were performed as triplicates.

### 4.4. Inhibition Experiments

Inhibition experiments for IC_50_ determination were performed for 1 min with the known inhibitor of organic cation transporter, decynium22, at the respective calculated *K*_m_-values of MPP (containing 2nM ^3^H MPP). The MPP uptake was cis-inhibited by following concentrations of decynium22: 1, 5, 10, 25, 50, 75, and 100 µM. Experiments were conducted on at least 2 separate days. On each day, all experiments were performed as triplicates.

For screening experiments, cis-inhibition was carried out in duplicate by measuring the uptake of the labeled probe substrate in the absence and presence of 10 µM or 100 µM of the respective pesticide or drug. Transporter- and vector transfected HEK293 cells were incubated for 1 min with 2 nM ^3^H-MPP or 1 µM ^14^C-metformin. Inhibitory effects in percent were calculated from net-uptake.

### 4.5. Determination of Protein Concentration

The cellular protein amount was determined using a method described by Bradford [48]. On each experimental day, six wells per cell line of an additional 24-well plate were analyzed in parallel to the transport experiments. Cell monolayers in 24-well plates were washed three times with 0.5 mL incubation buffer and afterwards stored at −20 °C. For protein determination, the plates were thawed and each well was incubated for lyses 30–60 min in 100 µL 1× lyses buffer (Promega, Manheim, Germany ). Cell lysate was filled up with ddH_2_O to 1 mL per well and mixed thoroughly. The protein determination was performed in 96-well plates (flat bottom; Sarstedt, Nümbrecht, Germany) in duplicate. BSA was used as standard for a calibration curve ranging from 50 to 300 µg/mL. A total of 20 µL of BSA standards or 20 µL sample (1:1 diluted in ddH_2_O) were mixed with 200 µL 1x Bradford reagent (Carl Roth) per well. After 10–20 min of incubation at room temperature, absorption was measured at 595 nm (Microplate Reader, Wallac Victor2 Perkin Elmer, Rodgau-Jügesheim, Germany). A standard curve was plotted from absorbance of 0–300 µg of BSA and the concentration of each test sample was determined using the standard curve.

### 4.6. Data Analysis

For the *K*_m_ calculation of MPP, the transporter-mediated uptake (pmol/mg protein/min) was plotted against MPP concentrations. The *K*_m_ and *V*_max_ values were obtained using SigmaPlot 13 by fitting the Michaelis–Menten equation V = V_max_*[S]/(K_m_ + [S]), where V refers to the rate of substrate transport, *V*_max_ refers to the maximum rate of substrate transport, [S] refers to the concentration of substrate, and Km is defined as the concentration of substrate at the half-maximal transport rate. The inhibitory effect I (%) was calculated according to the formula I(%) = 100 − (V_with inhibitor_*100/V_w/o inhibitor_), and, for the IC_50_ calculation of the inhibitor, the inhibitory effect I (%) was plotted against inhibitor concentrations and fitted using a 3-parameter Hill equation with I_max_ set to 100 using SigmaPlot 13.

## Figures and Tables

**Table 1:** Inhibitory effects of various cationic drugs to human, mouse and rat OCT1-mediated ^3^H-MPP uptake.

Drugs (15)	Type of Drug	Charge at pH 7.4	Inhibitory Effects (%)					
			hOCT1		mOct1		rOct1	
			10 µM	100 µM	10 µM	100 µM	10 µM	100 µM
Ketoconazol *	antifungal	82% uncharged 18% cation	47	83	46	74	43	75
Clonidine	hypertension	100% cation	73	83	72	82	62	80
Verapamil *	class IV antiarrhythmic agent	100% cation	39	82	29	64	29	64
Quinine *	anti malaria	100% cation	20	59	−7	50	−1	44
Elacridar	tumor drug resistance	100% cation	31	57	61	76	53	70
Quinidine *	class I antiarrhythmic agent	100% cation	13	53	−5	46	19	37
Procainamide *	class I antiarrhythmic agent	100% cation	10	43	−5	58	21	58
Ritonavir *	antiretroviral HIV	100% cation	17	39	8	−16	17	22
Ranitidine *	H2 histamine receptor antagonist	100% cation	10	38	−12	12	11	45
Zosuquidar	antineoplastic drug	37% uncharged 63% cation	2	20	−4	−23	0	4
Metformin *	type 2 diabetes	100% cation	11	13	12	9	−19	−4
Amiodarone *	class III antiarrhythmic agent	100% cation	9	10	−29	9	−25	1
Cimetidine *	H2 histamine receptor antagonist	75% uncharged 25% cation	8	9	−25	21	19	20
CyclosporinA *	immunsuppressant	100% cation	10	7	−27	6	−15	8
Reserpine	hypertension	70% uncharged 30% cation	7	6	−12	2	−17	12

* Asterisks show the compounds which are already published to interact with hOCT1 but not with all rodent Oct1 [8,19,20].

**Table 2:** Inhibitory effects of various pesticides to human, mouse and rat OCT1 mediated 3H-MPP uptake.

Pesticides (*n* = 9)	Type of Pesticide	Charge at pH 7.4	Inhibitory Effects (%)					
			hOCT1		mOct1		rOct1	
			10 µM	100 µM	10 µM	100 µM	10 µM	100 µM
Imazalil	fungicide	81% uncharged 19% cation	56	84	84	97	68	92
Propamocarb	fungicide	100% uncharged	9	49	29	63	24	57
Azoxystrobin	fungicide	100% uncharged	17	44	−2	49	0	42
Prochloraz	fungicide	100% cation	14	42	29	28	25	33
Atrazin	herbicide	100% uncharged	−8	15	−1	1	−65	−33
Amitraz	insecticide	100% cation	8	12	−23	21	−30	10
Glyphosat	herbicide	73% anion 27% ± charge	1	7	−9	22	−52	−19
Imidacloprid	insecticide	100% ± charge	4	1	23	−9	−5	−36
Paraquat *	herbicide	100% cation	0	−1	17	−9	1	−35

* The asterisk shows the compound which is already published to interact with hOCT1 but not with all rodent Oct1 [21].

**Table 3:** Inhibitory effects of various cationic drugs to hOCT1- and hOCT2-mediated ^3^H-MPP uptake and MATE1-mediated ^14^C-metformin uptake.

Drugs (14)	Inhibitory Effects (%)					
	hOCT1		hOCT2		hMATE1	
	10 µM	100 µM	10 µM	100 µM	10 µM	100 µM
Decynium22 *	87	90	42	90	76	97
Clonidine	73	83	69	80	24	60
Ketoconazol *	47	83	33	64	87	98
Verapamil *	39	82	35	43	44	82
Elacridar	31	57	−5	14	42	71
Quinidine *	13	53	0	24	11	24
Procainamide *	10	43	22	38	22	30
Ritonavir *	17	39	15	24	69	91
Ranitidine *	10	38	9	30	19	76
Zosuquidar	2	20	−19	14	23	65
Metformin *	11	13	10	6	5	36
Amiodarone *	9	10	9	19	1	12
CyclosporinA	10	7	−26	−27	18	6
Reserpine	7	6	3	8	72	83

* Asterisks show the compounds which are already published to interact with hOCT1 but not with all rodent Oct1 [8,19,20].

**Table 4:** Inhibitory effects of various pesticides to hOCT1- and hOCT2-mediated ^3^H-MPP uptake and MATE1-mediated ^14^C-metformin uptake.

Pesticides (9)	Inhibitory Effects (%)					
	OCT1		OCT2		MATE1	
	10 µM	100 µM	10 µM	100 µM	10 µM	100 µM
Imazalil	56	84	39	61	33	83
Propamocarb	9	49	24	54	22	25
Azoxystrobin	17	44	33	38	27	68
Prochloraz	14	42	32	33	7	70
Atrazin	−8	15	1	10	18	32
Amitraz	8	12	9	27	−11	12
Glyphosat	1	7	12	2	−1	14
Imidacloprid	4	1	−3	19	−8	29
Paraquat *	0	−1	−6	−4	3	4

* The asterisk shows the compound which is already published to interact with hOCT1 but not with all rodent Oct1 [21].
